# Drugs Modulating CD4+ T Cells Blood–Brain Barrier Interaction in Alzheimer’s Disease

**DOI:** 10.3390/pharmaceutics12090880

**Published:** 2020-09-16

**Authors:** Norwin Kubick, Patrick C. Henckell Flournoy, Ana-Maria Enciu, Gina Manda, Michel-Edwar Mickael

**Affiliations:** 1 Institute of Biochemistry, Molecular Cell Biology, University Clinic Hamburg-Eppendorf, 20251 Hamburg, Germany; n.kubick@uke.de; 2 PM Research Center, 20 Kaggeholm, Ekerö, 178 54 Stockholm, Sweden; pflourney@philomako.org; 3 Victor Babes National Institute of Pathology, 050096 București, Romania; ana.enciu@ivb.ro (A.-M.E.); contab@ivb.ro (G.M.); 4 Bevill Biomedical Sciences Research Building, UAB Birmingham, AL 35294-2170, USA; 5 Institute of Genetics and Animal Biotechnology of the Polish Academy of Sciences, ul. Postepu 36A, Jastrzębiec, 05-552 Magdalenka, Poland

**Keywords:** Alzheimer, blood brain barrier, CD4+ T cells, migration, Th17

## Abstract

The effect of Alzheimer’s disease (AD) medications on CD4+ T cells homing has not been thoroughly investigated. CD4+ T cells could both exacerbate and reduce AD symptoms based on their infiltrating subpopulations. Proinflammatory subpopulations such as Th1 and Th17 constitute a major source of proinflammatory cytokines that reduce endothelial integrity and stimulate astrocytes, resulting in the production of amyloid β. Anti-inflammatory subpopulations such as Th2 and Tregs reduce inflammation and regulate the function of Th1 and Th17. Recently, pathogenic Th17 has been shown to have a superior infiltrating capacity compared to other major CD4+ T cell subpopulations. Alzheimer’s drugs such as donepezil (Aricept), rivastigmine (Exelon), galantamine (Razadyne), and memantine (Namenda) are known to play an important part in regulating the mechanisms of the neurotransmitters. However, little is known about the effect of these drugs on CD4+ T cell subpopulations’ infiltration of the brain during AD. In this review, we focus on understanding the influence of AD drugs on CD4+ T cell subpopulation interactions with the BBB in AD. While current AD therapies improve endothelial integrity and reduce astrocytes activations, they vary according to their influence on various CD4+ T cell subpopulations. Donepezil reduces the numbers of Th1 but not Th2, Rivastigmine inhibits Th1 and Th17 but not Th2, and memantine reduces Th1 but not Treg. However, none of the current AD drugs is specifically designed to target the dysregulated balance in the Th17/Treg axis. Future drug design approaches should specifically consider inhibiting CD4+ Th17 to improve AD prognosis.

## 1. Who Are CD4+ T Cells?

**Figure 1:**
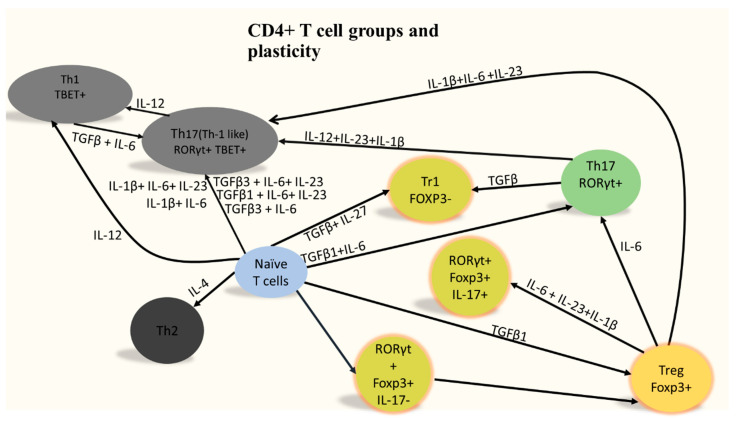
Naïve CD4+ T cells differentiation. After migrating from the thymus to the periphery, CD4+ T cells differentiate in the periphery into Th1, Th2, Th17, and Treg. CD4+ T cells are highly plastic with possibility of changing the fate of the cell based on its cytokines’ microenvironment. Tregs known for their ability to suppress proinflammatory CD4+ T cells can themselves be converted to highly pathogenic population under the action of IL-1, IL-6, and IL-23. Adapted from [4], copyright Bhaumik and Basu, 2017.

Peripheral CD4+ T cells constitute a major branch of adaptive immunity. These cells are produced in the bone marrow and emigrate to the thymus and then to the periphery where they continue to differentiate. Major peripheral CD4+ T cell subpopulations could be clustered into two main groups: (i) helper subpopulations such as Th1, Th2 and Th17; and (ii) regulatory subpopulations such as Tregs [1,2]. Each type of these subpopulations is classically governed by a master regulator. For example, Th1 is governed by *Tbx21*, while Th2 is controlled by *Gata3*. Th17 master regulator is *RoRc* and Treg is controlled by *FoxP3* expression. CD4+ T cell subpopulations could be characterized based on their cytokines production. For example, Th1 cells are known to produce IL-2 and IFN-γ as well as TNFα, making them proinflammatory and pathogenic, while Th2 cells produce IL-4, IL-5, and IL-13, giving them the ability to neutralize Th1 response. Th17 cells produce IL-17A and IL-17F. Tregs produce IL-10 and they can inhibit other CD4+ T cells. Th17 and Treg are controlled by a similar transcription network, thus they form the Th17/Treg axis [3]. It is important to note that CD4+ T cells could be programmable from one state to another according to their environmental circumstances. For example, Th17 could re-differentiate into a more pathogenic phenotype known as Th17(Th1-like) under the cytokine conditions IL-12, IL-23 and IL-1β (Figure 1).

## 2. CD4+ T Cells Infiltration Affects Alzheimer’s Disease Prognosis

Our current understanding of CD4+ T cells interaction with the brain during AD suggests that allowing anti-inflammatory CD4+ T cells infiltration while selectively limiting proinflammatory CD4+ T cells could enhance the disease prognosis. Exploiting CD4+ T cells infiltration in AD requires solving the CD4+ T cells paradox [5,6]. It was reported that these CD4+ T cells do not proliferate near the area of the plaques [7]. However, CD4+ T cells, which are reactive for amyloid β, produce proinflammatory cytokines, thus contributing to AD inflammatory response. Data also suggest that depletion of hippocampal T cells infiltration in tau-driven AD mouse models decreased spatial cognitive impairments [8]. Interestingly, the drug bexarotene [9] that causes apoptosis in T cells seemed to reverse the course of AD [10]. Conversely, mice lacking lymphocytes show a higher tendency of amyloid β plaques growth [11]. The solution to this paradox could be related to the variability of the impact of various T cell subpopulations on AD (Table 1). Alterations in the levels of various subpopulations of CD4+ T cells were identified in the Alzheimer patients’ blood. Overall, there was a rise in the frequency of CD4+ cells including FoxP3+ and Th17 subpopulations [12]. Specific T cell subpopulations could be performing an anti-inflammatory function by producing neurotrophic factors that protect neurons by stimulating the phagocytosis activity by microglia and thus help to reduce amyloid β deposition [13]. Th2 CD4+ T cells were reported to have a protective effect against AD [14] through their ability to induce the production of Aβ autoantibodies [15]. Moreover, reduction of regulatory T cells numbers shortened the time before APPPS1 mice showed a reduction in their cognitive abilities. Additionally, increasing the frequency of regulatory T cells by peripheral IL-2 injection augmented microglia numbers that are specifically targeting plaque and enhanced cognitive abilities in APPPS1 mice. Conversely, Th1 cells through the production of IFNγ had a negative impact on AD prognosis by augmenting microglial activation as well as increasing amyloid-β levels and exacerbating cognitive disabilities in an AD mouse model [16]. Proinflammatory evidence of Th17 function has been shown in AD through the upregulation of Th17-associated proinflammatory cytokines such as IL-17 and TNFα, which in turn are known to exacerbate β amyloid deposition and increase inflammation as well as reduce cognitive abilities [17]. Conversely, Treg plays an essential role in hindering the progression of AD [18] especially at the chronic stage. Taken together, these observations suggest that manipulation of specific CD4+ T cell subpopulations’ infiltration of the brain is critical for enhancing AD prognosis. In particular, the previous reports indicate that enhancing the infiltration of anti-inflammatory CD4+ T cells such as Treg while reducing the number of proinflammatory CD4+ T cells such as Th1 and Th17 could be a key element in improving AD inflammation; this hypothesis is also known to correct the Th17/Treg balance.

**Figure 2:**
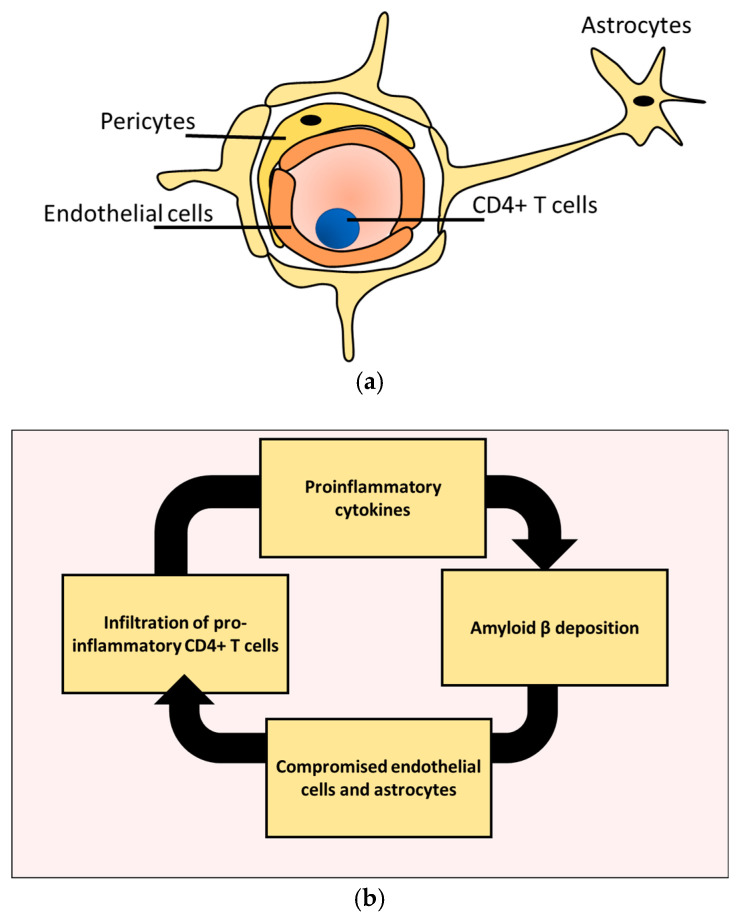
NVU is subjected to a vicious cycle of destruction in AD. (**a**) The main building blocks of the NVU unit includes endothelial cells, astrocytes, and pericytes. (**b**) Amyloid B deposition causes the BBB to lose its integrity, which in turn promotes infiltration of proinflammatory CD4+ T cells. Migrating CD4+ T cells produce proinflammatory cytokines that further increase the depiction of amyloid B.

Selective permission of anti-inflammatory CD4+ T cells migration to the brain while inhibiting proinflammatory CD4+ T cells requires controlling CD4+ T cells passage through the blood–brain barrier (BBB). The BBB is a protective boundary that regulate the passage of various substances to and from the brain, including lymphocytes [10]. The main building block of the BBB is known as a neurovascular unit (NUV). The NUV consists of endothelial cells, astrocytes, and pericytes (Figure 2a) [19]. Our current understanding of pericytes role is still fragmented and requires more systematic experiments. However, compelling examination suggests that endothelial cells and astrocytes are extensively influenced by AD pathologies. There seems to be a malicious feedback loop between Aβ buildup and endothelial cells as well as astrocytes damage during AD development [12,20]. This cycle could be one of the leading causes of the dysregulated effect of CD4+ T cells in AD (Figure 2b). This cycle is further exacerbated by proinflammatory cytokines produced by proinflammatory CD4+ T cells [21]. 

Therapeutic controlling of the interaction between CD4+ T cells and the BBB main components (i.e., endothelial cells and astrocytes) is vital in selective permission of anti-inflammatory CD4+ T cells while inhibiting proinflammatory CD4+ Th17 from entering the brain. This step will have a major impact on amyloid deposition, cognitive abilities maintenance, and reduction of brain inflammation in AD. In the remaining part of this review, we discuss the interactions between various CD4+ T cell subpopulations and endothelial cells as well as astrocytes, through the following steps. First, we compare the interactions between CD4+ T cells and endothelial cells as well as astrocytes under homeostasis and AD. Then, we summarize three groups of AD-associated compounds according to their effect on CD4+ T cell subpopulation interactions with the BBB: (i) classical AD drugs; (ii) current known compounds that can selectively inhibit CD4+ Th17 infiltration of the brain, and known to have a positive impact on AD prognosis; and (iii) compounds that are known to inhibit CD4+ Th17 and can be repurposed to treat AD.

## 3. CD4+ T Cells Interaction with Endothelial Cells under Homeostasis 

**Figure 3:**
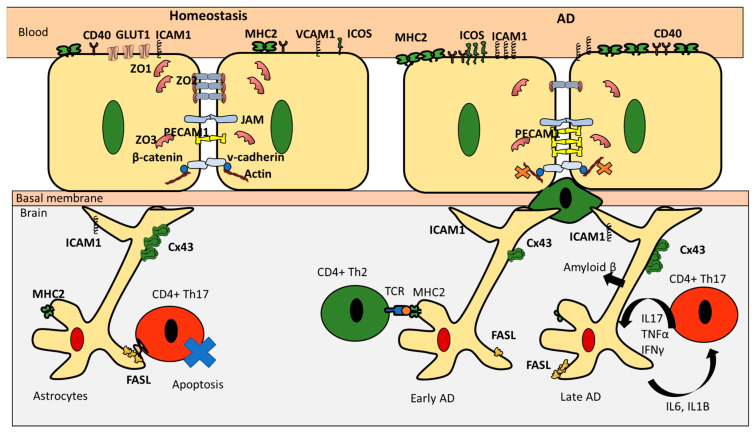
A comparison between CD4+ T cells interaction with the BBB in health and disease. During homeostasis, ICAM1 and VCAM1 are downregulated to deter adhesion. Occludin, ZO1, ZO2, and ZO3 are upregulated to main integrity. Cadherin is upregulated and PECAM1 is downregulated to prevent infiltration. This picture is mirrored in case of pathology, where the BBB loses its integrity, becomes more permeable, and increases the possibility of lymphocytes adhesion through upregulating VAM1 and ICAM1. During homeostasis, astrocytes upregulate Cx43 and FASL to inhibit CD4+ T cells infiltration. During the earlier stages of the disease, they downregulate Cx43 and present amyloid to Th2. However, during late phases of the disease, astrocytes themselves become a source of amyloid βand produce IL-6 and IL-1B which help recruit more pathogenic Th17 that produce proinflammatory cytokines forming a cycle of activation of pathological pathways.

It is generally accepted that the low infiltration of various CD4+ T cell subpopulations of the BBB during homeostasis (immune quiescence) could be attributed to the distinctive characteristics of the brain endothelial cells (BEC) such as: (i) elevated structural integrity; (ii) low rates of transcellular vesicular transport; and (iii) low expression levels of leukocyte adhesion molecules (Table 2) [22]. BEC structural integrity is supported by tight junction proteins and adherens junctions (AJ) [23]. Tight junctions are composed of claudin family members, occludin, junctional adhesion molecules (JAMs), and zonulae occludens (ZO1, ZO2, and ZO3) [24]. The main function of these proteins is to reduce paracellular diffusion. Interestingly, activated T cells express occludin [25]. Occludin is known to form homodimers [26]; whether occludin homodimers contribute to T cells modulation is still to be validated. The second element contributing to BEC structural integrity is adherens junctions (AJ) [27]. Adherens junctions such as cadherin proteins form homophilic bindings with cadherins expressed on neighboring cells through the intercellular space (intercellular cleft). KLRG1 is an inhibitory receptor expressed on effector CD4+ T cells (e.g., Th1, Th2, and Th17) as well as Treg. Several cadherins such as E, N, and R types have been pinpointed to be ligands for KLRG1 [28]. This observation suggests that cadherin could be diminishing T cells infiltration of the brain. AJs are secured into the actin cytoskeleton by the catenins (e.g., α, β, and γ catenin). Their principal task is to facilitate adhesion between neighboring cells. Interestingly, it was shown that β-catenin negatively regulates T cells activation [29]. Furthermore, the BBB is also characterized by low transcytosis rates of macromolecules, which are known to boost T cells proliferation, such as albumin, lipoprotein, and hormones (such as leptin) [30]. In the same venue, GLUT1 which, is known for its ability to regulate CD4+ T cells, is highly expressed in the BBB under homeostasis [31]. The ability of the BBB to modulate T cells infiltration could also be attributed to its low expression levels of leukocyte adhesion molecules (LAMs) such as E-selectin and ICAM1 [32]. Interestingly, it was reported that human brain microvascular endothelial cells (HBEC) under homeostasis also express low levels of MHC II, CD40, and ICOSL [33], thus suggesting a possible role in antigen presentation to CD4+ T cells (Figure 3). It is important to note however that different CD4+ T cells differ in their ability to cross the BBB, with Th17 showing a superior ability to infiltrate the brain under homeostatic conditions [34]. However, the reason behind this ability is still to be investigated.

**Figure 4:**
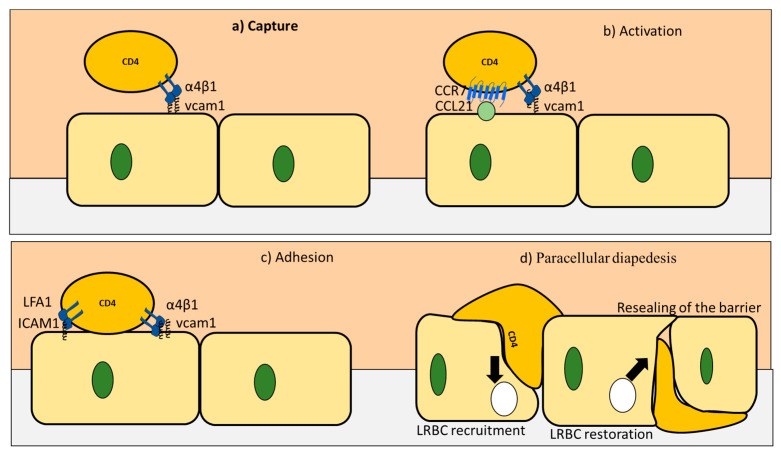
Four stages of extravasation of CD4+ T cells into the brain during health and disease. (**a**) In the capture stage, VCAM1 expressed on the endothelial cells binds to integrins expressed on CD4+ T cells. (**b**) After losing its speed, CD4+ T cells explore the surface of endothelial cells for ligands for their CCR7 receptor. (**c**) In the third stage known as adhesion, strong binds are formed using ICAM1 and VCAM1 from one side and LFA1 and α4β1 from the CD4+ T cells side. (**d**) Finally, paracellular diapedesis takes place, with cadherin downregulated, PECAM1 upregulated, and LRBC formed to store membrane proteins that takes an active part in the process. At the end of the process, LRBC is resorted, and sealing of the barrier takes place. Adapted from [35]. Copyright Springer, 2006.

During homeostasis as well as pathological conditions, CD4+ T cells infiltration of the BBB could be described through four stages [35] (Table 3 and Figure 4). Firstly, the capture stage is initiated by binding between VCAM1 expressed on the endothelial cells and VLA-4 (α4β1) expressed on CD4+ T cells. Secondly, CD4+ T cells probe the endothelial cells for chemokines ligands (e.g., CCL19 and CCL21) produced by endothelial cells or damaged neurons. If CCL19 and CCL21 are found, CCR7 expressed on the surface of CD4+ T cells binds to them [36] in what is known as the activation step. The activation step is followed by an adhesion phase. In this step, α4β1 and Lfa-1 expressed on the surface of CD4+ T cells bind to VCAM1 and ICAM1 on CD4+ T cells. These actions ensure firm attachment of CD4+ T cells to the endothelial cells surface, leading to the final phase of the dysbiosis [35]. CD4+ T cells prefer to pass between adjacent cells (paracellular) Paracellular diapedesis starts with VCAM1 and ICAM1 binding to the integrin, resulting in activation of the RAC1 and the SRC1 pathways, respectively. These two pathways phosphorylate VE-cadherin that is translocated from the junction. In the next step, PECAM1 expressed on the endothelial cells activates kinesin molecular motors. This in turn activates the lateral border recycling compartment (LBRC). The main function of the LBRC is to widen the space between the adjacent endothelial cells to facilitate CD4+ T cells passage to the brain. 

## 4. CD4+ T Cell Interactions with Astrocytes under Homeostasis 

Astrocytes play a critical role in maintaining BBB integrity and controlling CD4+ T cell migration under healthy conditions. Astrocytes are capable of polarizing T cells into both Th1 and Treg subtypes in vitro [37,38], indicating that, unlike endothelial cells, astrocytes may not preferentially interact with Th17. Furthermore, astrocytes express Cx43 which is a gap junction protein. Interestingly, Cx43 ko mice manifested a rise in CD4+ T cell infiltration of the brain as well as upregulation of MHC2, which was only found on APC [39]. Astrocytes express low levels of ICAM-1 under homeostasis, which is crucial for T cell homing (Table 4) [40]. Furthermore, Astrocytes can maintain homeostasis by inducing apoptosis in CD4+ T cell through a FASL mediated pathway [41] (Figure 3). Possible pathways by which astrocytes have been suggested to influence CD4+ T cell infiltration of the brain under homeostasis include: (i) PGE2 pathway; (ii) GABA; and (iii) LSAMP. astrocytes are known to produce PGE2 [42], which in turn can induce differentiation of regulatory T cells and suppress Th-cell proliferation [43]. Moreover, astrocytes could modulate T cells activity through a glutamate-GABA pathway. Astrocytes are known to release GABA [44]. GABA has an inhibitory role in autoimmune inflammation (Table 4) [45]. Moreover, LSAMP, an IgLON family member, is expressed by BBB astrocytes; deletions of LSAMP causes dysregulated reaction to new surroundings stressors, which in turn affect the immune system [46,47]. Overall, it is evident astrocytes preserve BBB cohesion and minimize CD4+ T cells infiltration to the brain during homeostasis.

## 5. CD4+ T Cells Endothelial Interactions in AD

Endothelial cells experience converse modifications that facilitate T cells migration in AD. These changes could be summarized in two aspects. The first is the reduction of the expression of the proteins responsible for the BBB high structural integrity. This is manifested in the reduction of TJ proteins, occludin, claudin5, and ZO1 (Figure 3 and Table 5) [20]. Additionally, β-amyloid seems to impair Wnt/β-catenin signaling at the blood–brain barrier [48]. Furthermore, endothelial GLUT1 deficiency results in disintegration of the BBB and speeding up Aβ deposition [49]. The second aspect is the increase in adhesion molecules accountable for CD4+ T cell homing such as VCAM1 and ICAM1 [7]. In addition, higher frequency of adhesion molecules known to be expressed on leukocytes such as E-selectin and Icam1 were also reported [50]. Besides, PECAM1 is elevated in AD patients [51]. This happens in addition to upregulation of MHC2, responsible for presenting antigens to infiltrating CD4+ T cells [52] These two aspects (i.e., reduction of molecules responsible for integrity and rise in the molecules responsible for recruitment) are responsible for the increase in infiltration of CD4+ T cells reported in AD [7,53]. It is important to note that Th17 cells have been reported to have superior abilities to cross the BBB during pathological conditions [34]. The reason behind Th17 exceptional abilities to travel through the BBB remains a topic of interesting research.

## 6. CD4+ T Cells and Astrocytes in AD

The dysregulated interaction between CD4+ T cells and astrocytes is an important factor in AD. The role of Astrocytes in AD is time dependent. At the early stages of the disease, astrocytes play a major role in clearing Aβ deposition. Additionally, astrocytes can also contribute to neuronal protection by restricting the access of Aβ deposits to them [38]. This could be one of the reasons behind astrocytes activation and accumulation during AD [38]. Interestingly, in newly formed plaques, it was found that Cx43 is downregulated, indicating that astrocytes are signaling for interaction with infiltrating CD4+ T cells. It was reported that astrocytes could present Aβ to Th2 cells that are specific for this antigen [54]. After they become activated, these Th2 cells acquire a regulatory ability and were shown to suppress proinflammatory CD4+ helper T cells such as Th1 and Th17 cells [54]. However, at later stages of overactivation and exposure to proinflammatory cytokines such as TNF-α + IFN-γ, astrocytes are stimulated to generate amyloidβ themselves [55]. Following this cycle, Aβ could upregulate the production of IL-6, IL-1β, and TNFα, as well as IFNγ, in astrocytes [56,57,58]. At that stage, astrocytes do not seem to selectively upregulate Th2 or Treg to induce anti-inflammatory mechanisms. On the contrary. IL-6 inhibits Th1 and Treg differentiation while increasing Th2 and Th17 differentiation (Figure 3 and Table 6) [59]. IL-6, IL-1β, and IL-23 could reprogram Treg to pathogenic Th17 (Figure 1). It was reported that, in older forming plaques, astrocytes upregulate Cx43. Since Cx43 is an inhibitory receptor for CD4+ T cells, these findings indicate that astrocytes could be trying to contain the damage of pathogenic CD4+ T cells at the later stages of the disease [60]. This hypothesis is further supported by the low ability of Aβ-specific Th1 or Th17 cells to cause an increase in MHC-II and CD86 levels in astrocytes. Furthermore, FASL pathway documented to be used by astrocytes to cause CD4+ T cells apoptosis is upregulated with the production of IL-1β and IL-6 by astrocytes [38]. There are reports that astrocytes exhibit higher expression levels of ICAM1; however, it was suggested that the increase in this cell adhesion molecules is to increase astrocytes interaction with microglia and not CD4+ T cells. Taken together, these reports indicate that targeting proinflammatory CD4+ T cells such as Th1 and Th17 could be the key to successful therapeutic strategies fighting AD. 

## 7. Effect of AD Drugs on CD4+ T Cells Proliferation and Differentiation

**Figure 5:**
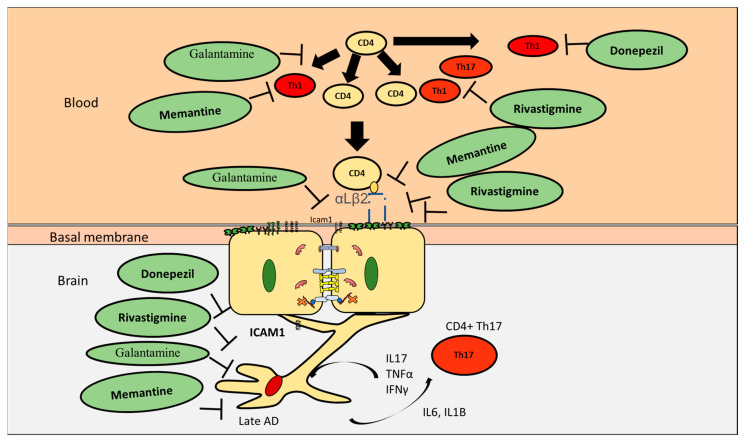
The effect of known AD drugs on CD4+ interaction with the BBB in AD. While donepezil, rivastigmine, and memantine are capable of inhibiting Th1, only rivastigmine was reported to inhibit Th17. Galantamine could inhibit proliferation of CD4+ T cells; however, its specificity towards CD4+ T cell subpopulations is still obscure. Nevertheless, all four drugs are capable of decreasing adhesion of CD4+ T cells to the surface of endothelial cells through downregulating ICAM1. They are also capable of decreasing astrocytes activation. However, their ability to target astrocytes Th17 interaction is still open for investigation.

In general, classical AD drugs seem to attenuate CD4+ T cells proliferation. Although Alzheimer drug therapy is a rich area of research, the main therapeutic strategies employed to fight AD are still cholinesterase inhibitors. Currently, there are four primary Alzheimer’s drugs: Donepezil, Rivastigmine, Galantamine, and memantine [61]. Donepezil, Rivastigmine, and Galantamine are cholinesterase inhibitors which act by inhibiting acetylcholinesterase, thus sustaining acetylcholine levels. The main hypothesis followed by this therapeutic approach is that high levels of Aβ can induce cholinergic cell toxicity and activation of cholinergic receptors can increase inter-cell communication [62]. Thus, by inhibiting cholinesterase, this strategy aims to preserve memory connections. Unfortunately, these drugs cannot stop the progression of dementia in AD patients. Donepezil is a safe drug with mild side effects [63]. It is reported to have some enhancements on cognitive abilities. However, no improvements were present on patient self-assessed quality of life. Donepezil is transported across the BBB by choline transporter [64]. In an interesting study, Jóźwik et al. found that soluble β-amyloids were unable to stimulate the proliferation of CD4+CD28+ T cells isolated from blood of patients who were administered donepezil [65]. It was shown that donepezil inhibits Th1 but not Th2 in EAE mice [66]. However, its effect on the Th17/Treg axis is unknown. Rivastigmine is another cholinergic inhibitor as it exercises its activity on acetylcholinesterase (AChE) and butyrylcholinesterase (BuChE). This drug is highly selective for the hippocampus and cortex. It also exerts positive influence on cognitive abilities [67]. Interestingly, it was shown that administration of rivastigmine resulted in reduction of T cell proliferation in AD patients’ blood [68]. However, rivastigmine was reported to induce a high incidence of gastrointestinal effects [69]. CD4+ T cells play a major role in the gastrointestinal homeostasis, thus whether these side effects are related to T cells inhibition is an open question. Interestingly, it was reported to inhibit Th1 and Th17 but not Th2 [70]. Galantamine is a competitive cholinesterase inhibitor that also modulates nicotinic acetylcholine receptors [71]. Unfortunately, it is associated with numerous side effects [72]. Galantamine seems to reduce T cells proliferation in certain disease such as diabetes [73]. However, its effect on T cell homing in AD is not yet known. Memantine is known to block the glutamate receptor NMDA. It has been shown that Aβ deposition as well as other hallmarks of Alzheimer’s disease can lead to overactivation of glutamatergic neurons. This in turn produces neurodegeneration [74]. Thus, memantine has proven useful in limiting neural degeneration. However, memantine also cannot stop the deposition of β amyloid. Furthermore, administration of memantine leads to a significant reduction of memory T cells (e.g., CD45RO+ CD4+) in the blood. This could be a double-edged sword as memantine can control unbalanced CD4+ T cells infiltration in AD, but may also increase the infection rate [75] Memantine can selectively inhibit Th1 but not Th2 or Treg [37,76] (Figure 5 and Table 7). However, its effect on Th17 is still not known.

## 8. Effect of Current AD Drugs on Endothelial Cells 

Current AD drugs seem to be able to reduce BBB permeability. It has been shown that donepezil amended endothelial cells permeability caused by TNFα. This hypothesis was supported by the upregulation of VE-cadherin, ZO1. The mechanism of action was reported to be through reducing the action of MMP9 and TIMP1, which are known to reduce the integrity of the BBB [77]. Galantamine was reported to significantly decrease ICAM1 expression on endothelial cells in the inflamed gut, thus inhibiting CD4+ adhesion to endothelial cells surface [78]. Rivastigmine was reported to increase transendothelial electrical resistance values, thus increasing BBB integrity and reducing permeability. It decreased the expression of ICAM and VCAM. It also preserved the expression of occludin and ZO1 (Figure 5 and Table 8) [79]. Although memantine uses a different mechanism of action, being an NMDA receptor, it was also reported to augment BBB stability through enhancing the expression of VE-cadherin and occludin expressed on endothelial cells through increasing MMP2 and not MMP9 [80]. It was also reported to inhibit ICAM1, thus decreasing the ability of CD4+ T cells adhesion [81]. Taken together, the above disused reports suggest that current AD drugs preserve the integrity of the BBB and indiscriminately inhibit CD4+ T cells adhesion to the endothelial cells.

## 9. Effect of AD Drugs on CD4+ T Cell Subpopulations Interactions with Astrocytes in AD

Currently used AD drugs seem to reduce anti-inflammatory CD4+ through decreasing astrocyte activation and pathogenic CD4+ T cells. For example, Donepezil was shown to reduce astrocytes activation and decrease their interaction [82]. Rivastigmine was also reported to decrease astrocytes activation by 50%. Galantamine attenuates amyloid-β deposition and astrocytes activation (Figure 5 and Table 9) [83]. Astrocytes stimulated with LPS or TNFα exhibited a rise in proinflammatory chemokines levels (such as CXCL10 and CCL20). Interestingly this proinflammatory response was eliminated by utilizing memantine [84]. Taken together, this section shows that current AD drugs could be reducing the activity of astrocytes. Decreasing astrocyte activation in turn reduces IL-1β and IL-6 production by astrocytes and decreases recruitment of pathogenic proinflammatory CD4+ T cells such as Th1 and Th17.

## 10. Alternatives for Existing Classical AD Drugs 

**Figure 6:**
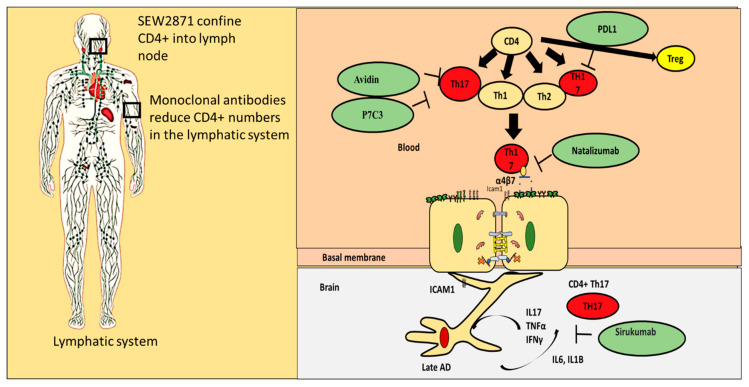
Repurposing old drugs to fight AD. Several strategies could be used to stop the progression of AD. SEW2871 could be used to confine CD4+ T cells into lymph nodes, while monoclonal antibodies could be used to reduce the number of CD4+ T cells. However, targeting Th17 through inhibition by avidin or P7C3 is more likely to decrease inflammation in the brain during AD. Sirukumab is an antibody against IL-6. Applying sirukumab could inhibit IL-6 production by astrocytes and hence decrease Th17 recruitment.

Existing classical AD drugs are known to decrease CD4+ T cells. However, currently, their effect on the Th17/Treg axis is lacking. There are multiple known alternatives for classical AD drugs that have a well-defined regulatory effect on the Th17/Treg axis (Figure 6 and Table 10). These drugs include Triptolide, which in known to inhibit Th17 and is considered to have a positive impact on AD prognosis. Quercetin is also known to inhibit Th17 and improve prognosis. Caffein, albumin, and insulin are also known to have a negative impact on Th17 but are positively correlated with Tregs.

## 11. Repurposing Drug Strategies That Can Regulate Proinflammatory CD4+ T Cells Interaction with the BBB

Several interactions between CD4+T cell subpopulations and the BBB could be exploited to manipulate CD4+ T cells migration to the brain. These interactions include regulating CD4+ T cell adhesion mechanisms, blocking differentiation of CD4+ T cells, confining CD4+ T cells to the lymph node, controlling CD4+ T cells fate, inhibiting IL-6 production, and inhibiting Th17 cells.

(i) Blocking proinflammatory CD4+ T cell subpopulations (e.g., Th1 and Th17) from entering the brain could reduce deposition and enhance cognitive function. Natalizumab is a monoclonal antibody against α4β7 and α4β1 integrins that are expressed on CD4+ T cells. This drug has proven potential in other neurogenerative diseases such as multiple sclerosis. It is important to note that one of the targets of Natalizumab (i.e., α4β7) is predominately expressed on proinflammatory CD4+ T cells and is less expressed on anti-inflammatory CD4+ T cells such as Tregs. [88]. Thus, Natalizumab is more selective for proinflammatory CD4+ T cells. Slavonic acid B has been reported to specifically inhibit Th1 infiltration of the brain in MS [89].
(ii) Confining CD4+ T cells to lymph nodes is an alternative strategy, which could be achieved through employing peripheral modulators such as Sphingosine-1-Phosphate receptor (S1PR). S1PR limits lymphocytes traffic and decreases their peripheral count, mainly by confining them into lymph nodes [90]. Several S1PR agonists (ponesimod, siponimod, amiselimod, and ozanimod) are currently tested in MS clinical trials. SEW2871 administration prevented cognitive abilities in Alzheimer’s rat model, indicating the S1P1R signaling pathway could be a new therapeutic target.
(iii) Monoclonal antibodies such as alemtuzumab decreased peripheral T cell count of both CD4+ T cells. However, its effect on AD patients is not yet known.
(iv) Other compounds that are capable of inhibiting Th17 include Avidin, Curcumin, Naringin, and P7C3
(v) One of the innovative approaches toward exploiting CD4+ T cells in treating AD is controlling CD4+ T cell fate; administration of PDL1 could help CD4+ T cells differentiate into Tregs and not into pathogenic Th17, which could be beneficial especially during the late stages of the disease.
(vi) Inhibiting IL-6 production by astrocytes production could lead to decrease in Th17 differentiation and recruitment; currently available anti-IL-6 drugs are sirukumab, olokizumab, elsilimomab, and siltuximab.

## 12. Conclusions

One of the effects of current drugs strategies is reducing inflammation by reducing CD4+ T cell migration to the brain in AD. CD4+ Th17 pathogenic T cells have superior capabilities of infiltrating the brain during pathological conditions. Although donepezil, rivastigmine, and memantine could reduce Th1 infiltration of the brain and decrease ICAM1 expression, only rivastigmine is targeting Th17. All the current drugs could reduce astrocytes activation, but none of them is specific toward decreasing IL-6 and IL-1β production by astrocytes. Continuous production of IL-6 and IL-1B by astrocytes not only increases recruitment of Th17, but also facilitates reprograming of anti-inflammatory Treg into Th17. This observation could be the reason behind the inability of current AD medications to stop the development of AD. Future perspectives of drug design should consider the detrimental effect Th17 might have on AD prognosis. Safe alternatives that could specifically target Th17 could be a key element in fighting AD.

## Figures and Tables

**Table 1:** Role of CD4+ T cells in AD.

CD4 Type	Event	Effect	Effect
Amyloid reactive CD4+ T cells	Produce proinflammatory cytokines	Prolonged inflammation in Alzheimer’s	Negative
CD4+ T cells	Depletion	Decreased impairment	Negative
CD4+ T cells	Apoptosis	Reverse AD	Positive
CD4+ T cells	Deficient mice	Larger growth of AD	Positive
Th2 CD4+ T cells	Induce of Aβ autoantibodies	Protective against AD	Positive
Treg CD4+ T cells	Increased microglia specific for plaques	Enhance cognitive abilities	Positive
Th1 CD4+ T cells	Produce IFNg, increase Aβ	Worsen cognitive abilities	Negative
Th17 CD4+ T cells	Produce proinflammatory cytokine	Increase inflammation in Alzheimer brain	Negative

**Table 2:** The effect of endothelial cells characteristics on CD4+ T cells migration in homeostasis.

Characteristics	Component	Function
High structural integrity	Tight junctions (occludin, JAMs, and zonulae occludens)	Restrict paracellular diffusion of CD4+ T cells
High structural integrity	Adherens junction (cadherins, catenin, and actin)	Mediate cell-cell adhesion to reduce CD4+ T cells infiltration
Low transcytosis rates of macromolecules promoting CD4+ T cells	albumin and leptin	Reduce T cells proliferation
High transcytosis rates of macromolecules reducing CD4+ T cells proliferation	GLUT1	Regulate T cells numbers
Low expression of leucocytes adhesion molecules	E-selectin and ICAM1	Reduce CD4+ T cells adhesion to the endothelial cells

**Table 3:** Various stages of CD4+ T cells infiltration of the BBB.

Stage	Component	Result of Stage Completion
Rolling stage	VCAM1 bind to VLA4 on CD4+ T cells	Weak bonds that reduce the speed of CD4+ T cells
Activation stage	e.g., CCL19 and CCL21 expressed on endothelial cells activate CD4+ T cells	CD4+ T cells are activated
Arrest stage	VCAM1 and ICAM bind to their ligands to mediate T cells arrest	CD4+ T cells attached to the endothelial cells
Diapedesis	V-cadherin gets phosphorylated	the catenin is released
	PECAM upregulation	Activate kinesin molecular motors
	LRBC trafficking	Expand the distance between endothelial cells

**Table 4:** Mechanism of action with which Astrocytes hinder CD4+ T cell migration in homeostasis.

Interaction	Effect
Astrocytes express high levels of Cx43	Reduce CD4+ T cells infiltration
Astrocytes express low levels of MHC2	Reduce CD4+ T cells activation
Astrocytes express low levels of ICAM	Reduce CD4+ T cells adhesion
Astrocytes activate FASL pathway	Induce apoptosis in CD4+ T cells
Astrocytes production of PGE2	Induce Treg and suppress Th1, Th12, Th17
Astrocytes production of GABA	Reduces Th-cell induced inflammation and hence migration

**Table 5:** The effect of endothelial cells characteristics on CD4+ T cells migration in AD.

Characteristics	Component	Function
Low structural integrity	Reduction in the expression of TJ proteins, occludin, claudin5, and ZO1	Reduce restriction of CD4+ T cells paracellular infiltration
Low structural integrity	Reduction in B catenein expression	Reduce adhesion between endothelial cells
Low transcytosis rates of macromolecules reducing CD4+ T cells proliferation	GLUT1	Reducing restriction of CD4+ T cells proliferation
Increase adhesion molecules	ICAM1 and VCAM1	Increase CD4+ T cells adhesion to the endothelial cells layer

**Table 6:** Mechanism of action with which Astrocytes increase CD4+ T cells migration in AD.

Interaction	Effect on CD4+ T Cells Migration
Astrocytes present Aβ to Th2	Th2 activation and acquire regulatory phenotype and inhibit Th1 and Th17
Astrocytes downregulate Cx43 (earlier stage)	Increase interaction with CD4+ T cells
Astrocytes produce IL-6	Activation of Th2, Th17 and inhibit Treg and Th1
Uptake TNF-α+IFN-γ produced by proinflammatory CD4+ T cells	Increase astrocytes activation and increase production of plaque
Astrocytes upregulate Cx43 (later stage)	decrease interaction with CD4+ T cells
Astrocytes slightly upregulate MHC2	Prevent further interaction with CD4+ T cells
Astrocytes upregulate FASL	Cause apoptosis

**Table 7:** Effect of Drugs on CD4 T cells proliferation and homing.

Drug	Effect CD4+ T Cells in AD
Donepezil	Inhibit Th1, promote Th2
Rivastigmine	Inhibit Th1, Th17 but not Th2
Galantamine	Decrease CD4+ T cells
Memantine	Inhibit Th1, promote Th2

**Table 8:** Effect of current AD drugs on endothelial cells of the BBB.

Drug	Effect Endothelial Cells
Donepezil	Increased BBB integrity, upregulated VE-cadherin and ZO1
Rivastigmine	Increased transendothelial electrical resistance, decreased ICAM1 and VAM1 and preserved occluding and ZO1
Galantamine	Significantly reduces ICAM1
Memantine	Inhibit ICAM1, increase BBB integrity, upregulate VE-cadherin and occludin

**Table 9:** Effect of drugs on astrocytes interaction with CD4+ T cells.

Drug	Effect on CD4+ T Cells Interaction with Astrocytes
Donepezil	Reduce astrocytes activation
Rivastigmine	Reduce astrocytes activation
memantine	Reduce astrocytes activation

**Table 10:** Compounds that enhance AD prognosis and inhibit CD4+ Th17.

Molecule	Effect on Th17/Treg Axis	Effect on AD
Triptolide	Inhibit Th17 cells [85]	Potential candidate for drug [86].
Caffeine (1,3,7-Trimethylpurine-2,6-dione)	Favors autoimmunity Treg and decreases cytokines needed for Th17 and Th1	Improve prognosis [87]
Albumin	Albumin functions as an inhibitor of T cell adhesion in vitro. Negatively correlated with Th17, positively correlated with Treg	Improve prognosis
Huperzine A	reduce lymphocyte proliferation and the secretion of proinflammatory cytokines	Choline esterase inhibitor
Insulin	Increase Treg	Improve prognosis
Ladostigil	reduce lymphocyte proliferation and the secretion of proinflammatory cytokines	a cholinesterase and monoamine oxidase inhibitor
Quercetin	Immunosuppressive, inhibit Th17	Improve prognosis

## Abbreviations

AD	Alzheimer’s disease
APC	Antigen presenting cells
BBB	Blood brain barrier
BEC	Brain Endothelial Cells
CNS	central nervous system
COX2	Cyclooxygenase-2
FoxP3	Forkhead Box P3
ICAM1	Intercellular Adhesion Molecule 1
IFNγ	Interferon gamma
IL-2	Interleukin 2
Il-6	Interleukin 6
NLRP3	PYD domains-containing protein 3
NSAIDs	Nonsteroidal anti-inflammatory drugs
PD-1	Programmed cell death protein 1
PECAM1	Platelet endothelial cell adhesion molecule 1
S1PR	Sphingosine-1-Phosphate receptor
TGF-β	Transforming growth factor beta
TJ	tight junctions
TNFα	Tumor necrosis factor alpha
VCAM1	Vascular cell adhesion protein 1
AChE	Acetylcholinesterase
ZO1	Zonula occludens-1
BuChE	Butyrylcholinesterase
GLUT1	Glucose transporter 1
MLCK	Myosin light-chain kinase
VE-cadherin	Vascular endothelial cell-specific cadherin

## References

[1] Mickael M.E., Bhaumik S., Basu R. (2020). Retinoid-related orphan receptor RORgammat in CD4(+) T-Cell-mediated intestinal homeostasis and inflammation. Am. J. Pathol..

[2] Allen J.E., Wynn T.A. (2011). Evolution of Th2 immunity: A rapid repair response to tissue destructive pathogens. PLoS Pathog..

[3] Ciofani M., Madar A., Galan C., Sellars M., Mace K., Pauli F., Agarwal A., Huang W., Parkhurst C.N., Muratet M. (2012). A validated regulatory network for Th17 cell specification. Cell.

[4] Bhaumik S., Basu R. (2017). Cellular and molecular dynamics of Th17 differentiation and its developmental plasticity in the intestinal immune response. Front. Immunol..

[5] Saresella M., Calabrese E., Marventano I., Piancone F., Gatti A., Alberoni M., Nemni R., Clerici M. (2011). Increased activity of Th-17 and Th-9 lymphocytes and a skewing of the post-thymic differentiation pathway are seen in Alzheimer’s disease. Brain Behav. Immun..

[6] Richartz-Salzburger E., Batra A., Stransky E., Laske C., Kohler N., Bartels M., Buchkremer G., Schott K. (2007). Altered lymphocyte distribution in Alzheimer’s disease. J. Psychiatr. Res..

[7] Ferretti M.T., Merlini M., Spani C., Gericke C., Schweizer N., Enzmann G., Engelhardt B., Kulic L., Suter T., Nitsch R.M. (2016). T-cell brain infiltration and immature antigen-presenting cells in transgenic models of Alzheimer’s disease-like cerebral amyloidosis. Brain Behav. Immun..

[8] Cai Z., Qiao P.F., Wan C.Q., Cai M., Zhou N.K., Li Q. (2018). Role of blood-brain barrier in Alzheimer’s disease. J. Alzheimers Dis..

[9] Tousi B. (2015). The emerging role of bexarotene in the treatment of Alzheimer’s disease: Current evidence. Neuropsychiatr. Dis. Treat..

[10] Alves S., Churlaud G., Audrain M., Michaelsen-Preusse K., Fol R., Souchet B., Braudeau J., Korte M., Klatzmann D., Cartier N. (2017). Interleukin-2 improves amyloid pathology, synaptic failure and memory in Alzheimer’s disease mice. Brain.

[11] Bryson K.J., Lynch M.A. (2016). Linking T cells to Alzheimer’s disease: From neurodegeneration to neurorepair. Curr. Opin. Pharmacol..

[12] Mietelska-Porowska A., Wojda U. (2017). T Lymphocytes and inflammatory mediators in the interplay between brain and blood in Alzheimer’s disease: Potential Pools of New Biomarkers. J. Immunol. Res..

[13] Baruch K., Rosenzweig N., Kertser A., Deczkowska A., Sharif A.M., Spinrad A., Tsitsou-Kampeli A., Sarel A., Cahalon L., Schwartz M. (2015). Breaking immune tolerance by targeting Foxp3(+) regulatory T cells mitigates Alzheimer’s disease pathology. Nat. Commun..

[14] Haddad-Tovolli R., Dragano N.R.V., Ramalho A.F.S., Velloso L.A. (2017). Development and function of the blood-brain barrier in the context of metabolic control. Front. Neurosci..

[15] Dionisio-Santos D.A., Olschowka J.A., O’Banion M.K. (2019). Exploiting microglial and peripheral immune cell crosstalk to treat Alzheimer’s disease. J. Neuroinflamm..

[16] Browne T.C., McQuillan K., McManus R.M., O’Reilly J.A., Mills K.H., Lynch M.A. (2013). IFN-gamma Production by amyloid beta-specific Th1 cells promotes microglial activation and increases plaque burden in a mouse model of Alzheimer’s disease. J. Immunol..

[17] Cristiano C., Volpicelli F., Lippiello P., Buono B., Raucci F., Piccolo M., Iqbal A.J., Irace C., Miniaci M.C., Perrone Capano C. (2019). Neutralization of IL-17 rescues amyloid-beta-induced neuroinflammation and memory impairment. Br. J. Pharmacol..

[18] Dansokho C., Ait Ahmed D., Aid S., Toly-Ndour C., Chaigneau T., Calle V., Cagnard N., Holzenberger M., Piaggio E., Aucouturier P. (2016). Regulatory T cells delay disease progression in Alzheimer-like pathology. Brain.

[19] Sagare A.P., Bell R.D., Zhao Z., Ma Q., Winkler E.A., Ramanathan A., Zlokovic B.V. (2013). Pericyte loss influences Alzheimer-like neurodegeneration in mice. Nat. Commun..

[20] Yamazaki Y., Kanekiyo T. (2017). Blood-brain barrier dysfunction and the pathogenesis of Alzheimer’s disease. Int. J. Mol. Sci..

[21] Alasmari F., Alshammari M.A., Alasmari A.F., Alanazi W.A., Alhazzani K. (2018). Neuroinflammatory cytokines induce amyloid beta neurotoxicity through modulating amyloid precursor protein levels/metabolism. Biomed. Res. Int..

[22] Haseloff R.F., Dithmer S., Winkler L., Wolburg H., Blasig I.E. (2015). Transmembrane proteins of the tight junctions at the blood-brain barrier: Structural and functional aspects. Semin. Cell Dev. Biol..

[23] Greene C., Campbell M. (2016). Tight junction modulation of the blood brain barrier: CNS delivery of small molecules. Tissue Barriers.

[24] Sonar S.A., Lal G. (2018). Blood-brain barrier and its function during inflammation and autoimmunity. J. Leukoc. Biol..

[25] Paul D., Baena V., Ge S., Jiang X., Jellison E.R., Kiprono T., Agalliu D., Pachter J.S. (2016). Appearance of claudin-5(+) leukocytes in the central nervous system during neuroinflammation: A novel role for endothelial-derived extracellular vesicles. J. Neuroinflamm..

[26] Cummins P.M. (2012). Occludin: One protein, many forms. Mol. Cell Biol..

[27] Tietz S., Engelhardt B. (2015). Brain barriers: Crosstalk between complex tight junctions and adherens junctions. J. Cell Biol..

[28] Banh C., Fugere C., Brossay L. (2009). Immunoregulatory functions of KLRG1 cadherin interactions are dependent on forward and reverse signaling. Blood.

[29] Ma J., Wang R., Fang X., Sun Z. (2012). β-catenin/TCF-1 pathway in T cell development and differentiation. J. Neuroimmune Pharmacol..

[30] Fernandez-Riejos P., Najib S., Santos-Alvarez J., Martin-Romero C., Perez-Perez A., Gonzalez-Yanes C., Sanchez-Margalet V. (2010). Role of leptin in the activation of immune cells. Mediat. Inflamm..

[31] Macintyre A.N., Gerriets V.A., Nichols A.G., Michalek R.D., Rudolph M.C., Deoliveira D., Anderson S.M., Abel E.D., Chen B.J., Hale L.P. (2014). The glucose transporter Glut1 is selectively essential for CD4 T cell activation and effector function. Cell Metab..

[32] Chow B.W., Gu C. (2015). The molecular constituents of the blood-brain barrier. Trends Neurosci..

[33] Wheway J., Obeid S., Couraud P.O., Combes V., Grau G.E. (2013). The brain microvascular endothelium supports T cell proliferation and has potential for alloantigen presentation. PLoS ONE.

[34] Nishihara H., Soldati S., Mossu A., Rosito M., Rudolph H., Muller W.A., Latorre D., Sallusto F., Sospedra M., Martin R. (2020). Human CD4(+) T cell subsets differ in their abilities to cross endothelial and epithelial brain barriers in vitro. Fluids Barriers CNS.

[35] Engelhardt B. (2006). Molecular mechanisms involved in T cell migration across the blood-brain barrier. J. Neural Transm..

[36] Noor S., Wilson E.H. (2012). Role of C-C chemokine receptor type 7 and its ligands during neuroinflammation. J. Neuroinflammation.

[37] Beurel E., Harrington L.E., Buchser W., Lemmon V., Jope R.S. (2014). Astrocytes modulate the polarization of CD4+ T cells to Th1 cells. PLoS ONE.

[38] Xie L., Yang S.H. (2015). Interaction of astrocytes and T cells in physiological and pathological conditions. Brain Res..

[39] Boulay A.C., Mazeraud A., Cisternino S., Saubamea B., Mailly P., Jourdren L., Blugeon C., Mignon V., Smirnova M., Cavallo A. (2015). Immune quiescence of the brain is set by astroglial connexin 43. J. Neurosci..

[40] Frohman E.M., Frohman T.C., Dustin M.L., Vayuvegula B., Choi B., Gupta A., van den Noort S., Gupta S. (1989). The induction of intercellular adhesion molecule 1 (ICAM-1) expression on human fetal astrocytes by interferon-λ, tumor necrosis factor α, lymphotoxin, and interleukin-1: Relevance to intracerebral antigen presentation. J. Neuroimmunol..

[41] Gimsa U., Mitchison N.A., Brunner-Weinzierl M.C. (2013). Immune privilege as an intrinsic CNS property: Astrocytes protect the CNS against T-cell-mediated neuroinflammation. Mediators Inflamm..

[42] MacVicar B.A., Newman E.A. (2015). Astrocyte regulation of blood flow in the brain. Cold Spring Harb. Perspect. Biol..

[43] Sreeramkumar V., Fresno M., Cuesta N. (2012). Prostaglandin E2 and T cells: Friends or foes?. Immunol. Cell Biol..

[44] Covelo A., Araque A. (2018). Neuronal activity determines distinct gliotransmitter release from a single astrocyte. Elife.

[45] Bhat R., Axtell R., Mitra A., Miranda M., Lock C., Tsien R.W., Steinman L. (2010). Inhibitory role for GABA in autoimmune inflammation. Proc. Natl. Acad. Sci. USA.

[46] Dhabhar F.S. (2018). The short-term stress response—Mother nature’s mechanism for enhancing protection and performance under conditions of threat, challenge, and opportunity. Front. Neuroendocrinol..

[47] Kubick N., Brosamle D., Mickael M.E. (2018). Molecular evolution and functional divergence of the IgLON family. Evol. Bioinform. Online.

[48] Vallee A., Lecarpentier Y. (2016). Alzheimer disease: Crosstalk between the canonical Wnt/Beta-Catenin pathway and PPARs Alpha and Gamma. Front. Neurosci..

[49] Winkler E.A., Nishida Y., Sagare A.P., Rege S.V., Bell R.D., Perlmutter D., Sengillo J.D., Hillman S., Kong P., Nelson A.R. (2015). GLUT1 reductions exacerbate Alzheimer’s disease vasculo-neuronal dysfunction and degeneration. Nat. Neurosci..

[50] Muller W.A. (2011). Mechanisms of leukocyte transendothelial migration. Annu. Rev. Pathol..

[51] Zenaro E., Piacentino G., Constantin G. (2017). The blood-brain barrier in Alzheimer’s disease. Neurobiol. Dis..

[52] Lopes Pinheiro M.A., Kamermans A., Garcia-Vallejo J.J., van Het Hof B., Wierts L., O’Toole T., Boeve D., Verstege M., van der Pol S.M., van Kooyk Y. (2016). Internalization and presentation of myelin antigens by the brain endothelium guides antigen-specific T cell migration. Elife.

[53] Wennstrom M., Nielsen H.M. (2012). Cell adhesion molecules in Alzheimer’s disease. Degener. Neurol. Neuromuscul. Dis..

[54] McQuillan K., Lynch M.A., Mills K.H. (2010). Activation of mixed glia by Abeta-specific Th1 and Th17 cells and its regulation by Th2 cells. Brain Behav. Immun..

[55] Zhao J., O’Connor T., Vassar R. (2011). The contribution of activated astrocytes to Abeta production: Implications for Alzheimer’s disease pathogenesis. J. Neuroinflamm..

[56] Chun H., Marriott I., Lee C.J., Cho H. (2018). Elucidating the interactive roles of glia in Alzheimer’s disease using established and newly developed experimental models. Front. Neurol..

[57] Li C., Zhao R., Gao K., Wei Z., Yin M.Y., Lau L.T., Chui D., Yu A.C. (2011). Astrocytes: Implications for neuroinflammatory pathogenesis of Alzheimer’s disease. Curr. Alzheimer Res..

[58] Bhat R., Crowe E.P., Bitto A., Moh M., Katsetos C.D., Garcia F.U., Johnson F.B., Trojanowski J.Q., Sell C., Torres C. (2012). Astrocyte senescence as a component of Alzheimer’s disease. PLoS ONE.

[59] Basu R., Hatton R.D., Weaver C.T. (2013). The Th17 family: Flexibility follows function. Immunol. Rev..

[60] Xing L., Yang T., Cui S., Chen G. (2019). Connexin hemichannels in astrocytes: Role in CNS disorders. Front. Mol. Neurosci..

[61] Melnikova I. (2007). Therapies for Alzheimer’s disease. Nat. Rev. Drug Discov..

[62] Kar S., Slowikowski S.P., Westaway D., Mount H.T. (2004). Interactions between beta-amyloid and central cholinergic neurons: Implications for Alzheimer’s disease. J. Psychiatry Neurosci..

[63] Seltzer B. (2005). Donepezil: A review. Expert Opin. Drug Metab. Toxicol..

[64] Banks W.A. (2012). Drug delivery to the brain in Alzheimer’s disease: Consideration of the blood-brain barrier. Adv. Drug Deliv. Rev..

[65] Jozwik A., Landowski J., Bidzan L., Fulop T., Bryl E., Witkowski J.M. (2012). Beta-amyloid peptides enhance the proliferative response of activated CD4CD28 lymphocytes from Alzheimer disease patients and from healthy elderly. PLoS ONE.

[66] Jiang Y., Zou Y., Chen S., Zhu C., Wu A., Liu Y., Ma L., Zhu D., Ma X., Liu M. (2013). The anti-inflammatory effect of donepezil on experimental autoimmune encephalomyelitis in C57 BL/6 mice. Neuropharmacology.

[67] Onor M.L., Trevisiol M., Aguglia E. (2007). Rivastigmine in the treatment of Alzheimer’s disease: An update. Clin. Interv. Aging.

[68] Busse S., Steiner J., Glorius S., Dobrowolny H., Greiner-Bohl S., Mawrin C., Bommhardt U., Hartig R., Bogerts B., Busse M. (2015). VGF expression by T lymphocytes in patients with Alzheimer’s disease. Oncotarget.

[69] Darreh-Shori T., Jelic V. (2010). Safety and tolerability of transdermal and oral rivastigmine in Alzheimer’s disease and Parkinson’s disease dementia. Expert Opin. Drug Saf..

[70] Nizri E., Irony-Tur-Sinai M., Faranesh N., Lavon I., Lavi E., Weinstock M., Brenner T. (2008). Suppression of neuroinflammation and immunomodulation by the acetylcholinesterase inhibitor rivastigmine. J. Neuroimmunol..

[71] Raskind M.A., Peskind E.R., Wessel T., Yuan W. (2000). Galantamine in AD: A 6-month randomized, placebo-controlled trial with a 6-month extension. The Galantamine USA-1 Study Group. Neurology.

[72] Wilkinson D., Murray J. (2001). Galantamine: A randomized, double-blind, dose comparison in patients with Alzheimer’s disease. Int. J. Geriatr. Psychiatry.

[73] Hanes W.M., Olofsson P.S., Kwan K., Hudson L.K., Chavan S.S., Pavlov V.A., Tracey K.J. (2015). Galantamine attenuates type 1 diabetes and inhibits anti-insulin antibodies in nonobese diabetic mice. Mol. Med..

[74] Danysz W., Parsons C.G. (2012). Alzheimer’s disease, beta-amyloid, glutamate, NMDA receptors and memantine-searching for the connections. Br. J. Pharmacol..

[75] Lowinus T., Bose T., Busse S., Busse M., Reinhold D., Schraven B., Bommhardt U.H. (2016). Immunomodulation by memantine in therapy of Alzheimer’s disease is mediated through inhibition of Kv1.3 channels and T cell responsiveness. Oncotarget.

[76] Kahlfuss S., Simma N., Mankiewicz J., Bose T., Lowinus T., Klein-Hessling S., Sprengel R., Schraven B., Heine M., Bommhardt U. (2014). Immunosuppression by N-methyl-D-aspartate receptor antagonists is mediated through inhibition of Kv1.3 and KCa3.1 channels in T cells. Mol. Cell Biol..

[77] Tang X., Di X., Liu Y. (2017). Protective effects of Donepezil against endothelial permeability. Eur. J. Pharmacol..

[78] Wazea S.A., Wadie W., Bahgat A.K., El-Abhar H.S. (2018). Galantamine anti-colitic effect: Role of alpha-7 nicotinic acetylcholine receptor in modulating Jak/STAT3, NF-kappaB/HMGB1/RAGE and p-AKT/Bcl-2 pathways. Sci. Rep..

[79] Liu R., Zhang T.-T., Wu C.-X., Lan X., Du G.-H. (2011). Targeting the neurovascular unit: Development of a new model and consideration for novel strategy for Alzheimer’s disease. Brain Res. Bull..

[80] Liu Y., Huang Y., Xu Y., Qu P., Wang M. (2018). Memantine protects against ischemia/reperfusion-induced brain endothelial permeability. IUBMB Life.

[81] Wang F., Zou Z., Gong Y., Yuan D., Chen X., Sun T. (2017). Regulation of human brain microvascular endothelial cell adhesion and barrier functions by memantine. J. Mol. Neurosci..

[82] Gonzalez-Reyes R.E., Nava-Mesa M.O., Vargas-Sanchez K., Ariza-Salamanca D., Mora-Munoz L. (2017). Involvement of astrocytes in Alzheimer’s disease from a neuroinflammatory and oxidative stress perspective. Front. Mol. Neurosci..

[83] Wu Z., Zhao L., Chen X., Cheng X., Zhang Y. (2015). Galantamine attenuates amyloid-beta deposition and astrocyte activation in APP/PS1 transgenic mice. Exp. Gerontol..

[84] Skowronska K., Obara-Michlewska M., Zielinska M., Albrecht J. (2019). NMDA receptors in astrocytes: In search for roles in neurotransmission and astrocytic homeostasis. Int. J. Mol. Sci..

[85] Wang Y., Jia L., Wu C.Y. (2008). Triptolide inhibits the differentiation of Th17 cells and suppresses collagen-induced arthritis. Scand. J. Immunol..

[86] Wang Q., Xiao B., Cui S., Song H., Qian Y., Dong L., An H., Cui Y., Zhang W., He Y. (2014). Triptolide treatment reduces Alzheimer’s disease (AD)-like pathology through inhibition of BACE1 in a transgenic mouse model of AD. Dis. Model. Mech..

[87] Eskelinen M.H., Kivipelto M. (2010). Caffeine as a protective factor in dementia and Alzheimer’s disease. J. Alzheimers Dis..

[88] Stenner M.P., Waschbisch A., Buck D., Doerck S., Einsele H., Toyka K.V., Wiendl H. (2008). Effects of natalizumab treatment on Foxp3+ T regulatory cells. PLoS ONE.

[89] Dong Z., Ma D., Gong Y., Yu T., Yao G. (2016). Salvianolic acid B ameliorates CNS autoimmunity by suppressing Th1 responses. Neurosci. Lett..

[90] Perez-Jeldres T., Tyler C.J., Boyer J.D., Karuppuchamy T., Yarur A., Giles D.A., Yeasmin S., Lundborg L., Sandborn W.J., Patel D.R. (2019). Targeting cytokine signaling and lymphocyte traffic via small molecules in inflammatory bowel disease: JAK inhibitors and S1PR agonists. Front. Pharmacol..

